# HIV-1 Tat interaction with Dicer: requirement for RNA

**DOI:** 10.1186/1742-4690-3-95

**Published:** 2006-12-20

**Authors:** Yamina Bennasser, Kuan-Teh Jeang

**Affiliations:** 1 Molecular Virology Section, Laboratory of Molecular Microbiology, National Institute of Allergy and Infectious Diseases, National Institutes of Health, Bethesda, Maryland 20892-0460, USA

## Abstract

Dicer is an RNase III which processes two classes of cellular small RNAs: the microRNAs (miRNA) and short interfering RNAs (siRNA). Previously, we observed that over-expressed HIV-1 Tat protein can suppress the processing of small RNAs inside cells. Here, we have investigated the requirements for Tat interaction with Dicer. We report that Tat-Dicer interaction depends on RNA, requires the helicase domain of Dicer, and is independent of Tat's transactivation domain.

## Findings

The cell's RNA interference (RNAi) machinery is involved in either the inhibition of gene expression by sequence-specific cleavage of mRNAs or translational silencing of targeted RNAs [1-3]. One component of the RNAi machinery is Dicer, an ATP-dependent RNase III, which processes two classes of small RNAs: microRNA (miRNA) and short interfering RNA (siRNA) [4]. In the cytoplasm, Dicer recognizes a pre-miRNA, a short hairpin structure containing an imperfect stem, and generates small mature miRNA duplexes of 21 to 25 nucleotides. Pre-miRNAs originate from nuclear pri-miRNAs which are RNA polymerase II transcribed cellular transcripts that are processed by another RNase III protein, Drosha. Processed pre-miRNAs are shuttled from the nucleus into the cytoplasm by the exportin-5 protein.

In the cytoplasm, a Dicer-miRNA complex recognizes a dsRNA binding protein called TRBP (for "TAR RNA binding protein"). TRBP connects Dicer-miRNA into the RNA induced silencing complex (RISC) through interaction with the argonaute 2 (Ago-2) protein [5,6]. Within RISC, one strand of the miRNA duplex is retained and serves as a guide RNA for base-complementary recognition of RNA-targets. It is currently thought that miRNA-RISC captures target transcripts through guide RNA – target RNA base complementarity; the target RNA is subsequently translationally silenced by sequestration into ribosome-free cytoplasmic compartments called processing bodies (P-bodies) [7,8]. Because miRNA-RISC mediated translational inhibition of target mRNA does not require perfect miRNA-mRNA complementarity, one miRNA is in principle capable of silencing the translation of more than one hundred cellular transcripts [9]. In this respect, eucaryotic miRNAs are reasoned to be potentially capable of regulating the protein expression of more than 30 % of cellular genes [10]. In addition to its role in miRNA processing, Dicer also recognizes dsRNAs which originate from viruses, transgenes or transposons and cleaves them into small duplexes of 18 to 21 nucleotides called siRNA [11]. Like miRNAs, one strand of siRNAs is incorporated into RISC to be used as a guide sequence [12]. siRNA-guided RISC requires perfect complementarity with target mRNAs to promote not translational silencing but ribonuclease-mediated degradation of targeted transcripts.

It has been proposed that mammalian cells may use RNAi as a defense against infection by viruses [13-15]. However, because most viral infections seem to progress efficiently in cells, one surmises that many viruses have developed stratagems to evade or suppress the cell's RNAi machinery [13,16,17]. Several extant observations are consistent with an RNAi thrust-and-parry interplay between the cell and the virus. For example, HIV-1 infection appears to down regulate the cell's miRNA processing [18], perhaps by encoding a partially effective suppressors of RNAi processing [16,19]. HIV-1 can also mutate its coding sequence to evade base-pair complementarity driven RNAi [20]. Additionally, HIV-1 can encode small si-/mi- RNA-like decoys, such as TAR RNA, which can squelch TRBP making this critical factor unavailable for authentic si-/mi- RNA processing [21,22].

We previously suggested that the HIV-1 Tat protein can act to suppress si-/mi- RNA processing [19]. In our experiments, over-expression of Tat in cells reduced the efficiency of shRNA-mediated RNAi. We also noted that Tat can inhibit Dicer activity *in vitro*. This activity of Tat was separate from its trans-activation function since a trans-activation inactive TatK41A mutant still retained suppression of RNA silencing (SRS) activity [19]. Here, we characterized the requirements for over-expressed Tat to interact with Dicer.

### Tat interaction with Dicer requires RNA

**Figure 1:**
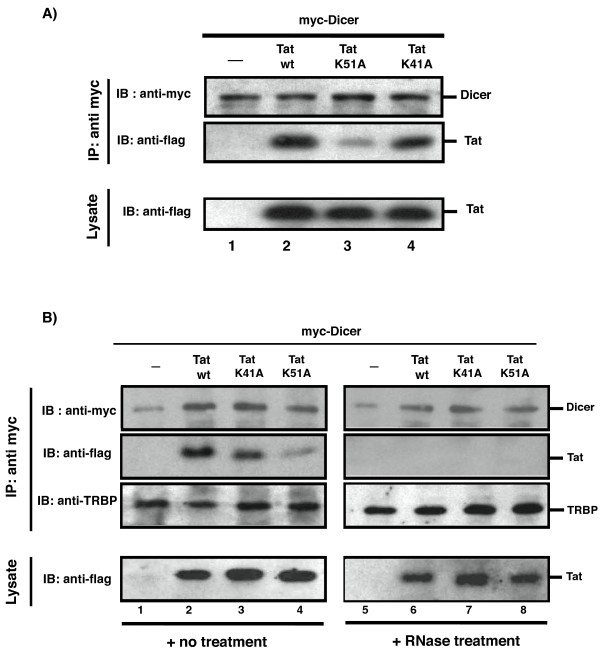
**Tat co-immunoprecipitation with Dicer requires RNA**. **A) **293T cells were transfected with pcDNA-Dicer-myc (lane 1) or cotransfected with pcDNA-Dicer-myc and pcDNA-wtTat-flag (lane 2) or Tat point mutants, TatK41A or TatK51A (lane 3 and 4). 48 hours later, cell lysates were immunoprecipitated with anti-myc beads overnight at 4°C. Dicer-immunoprecipitates were assessed by Western blotting using anti-myc (top panel) and co-immunoprecipitated Tat was detected using anti-flag (middle panel). As a control, the amounts of wt Tat and Tat mutants were verified in total cell lysates (lower panel). **B) **Co-immunoprecipitation analyses of transfected samples after no treatment (lane 1 to 4) or treatment with 50 μg/ml of RNase A (lanes 5 to 8). In addition to immunoblotting for Dicer and Tat, presence of TRBP in the immunoprecipitations was also analyzed.

We assayed Tat interaction with Dicer by transfecting 293T cells with myc-tagged Dicer (pDicer-myc) in the absence or presence of flag-tagged Tat (pTat-flag) (Figure 1). Cell extracts were immunoprecipitated with anti-myc beads, and analyzed by Western blotting. As shown in figure 1, Tat co-immunoprecipitated (co-IP) with Dicer (lane 2). To assess better Tat/Dicer interaction, we conducted the co-IP using two Tat point-mutants. The TatK51A mutant previously was found to have little suppressive effect on Dicer activity while being proficient for viral transactivation; the TatK41A mutant did moderate Dicer activity while being deficient in Tat's transcriptional transactivation activity.

We expressed Tat, TatK51A and TatK41A comparably (Figure 1A, lower panel), and we also expressed myc-Dicer equally in each of the transfections (Figure 1A, upper panel). When Dicer was immunoprecipitated, we found that the recovery of the various Tat proteins was different. Tat K41A and Tat co-immunoprecipitated similarly with Dicer (Figure 1A, lanes 2 and 4); however, Tat K51A reproducibly co-immunoprecipitated less effectively (Figure 1A, lane 3). These results suggest that the association between Tat and Dicer as assayed by co-IP correlates with the ability of the former to moderate the activity of the latter.

Because Tat and Dicer are both RNA-binding proteins, we wondered next if their interaction required RNA. To address this question, lysates from cells transfected with myc-Dicer and Tat proteins were divided into two groups prior to immunoprecipitation. One group was treated with RNase A while the other group was not (Figure 1B). Without RNase treatment (Figure 1B, lanes 1 to 4), Tat and TatK41A interacted well with Dicer while TatK51A did less well; however, after RNase treatment, none of the Tat proteins was able to co-immunoprecipitate with Dicer (Figure 1B, lanes 6, 7 and 8). As a control, the amounts of the Tat proteins in the lysates were verified to be unchanged after RNase treatment (Figure 1B, right lower panel). Furthermore, TRBP, whose interaction with Dicer is RNA independent [23], co-immunoprecipitated with Dicer comparably regardless of RNase treatment (compare anti-TRBP, Figure 1B left to right). Hence, Tat and TRBP interact differently with Dicer; the former requires RNA while the latter does not. It remains not known whether a specific form of RNA (i.e. pre-miRNA) or general cellular RNAs suffice to mediate Dicer and Tat interaction. This requirement needs to be investigated further.

### Dicer's helicase domain is required for interaction with Tat

**Figure 2:**
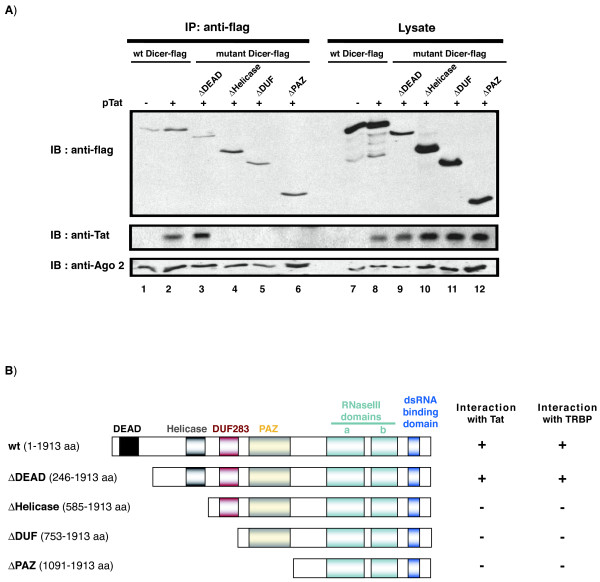
**Dicer's helicase domain is required for co-immunoprecipitating Tat**. **A) **Co-immunoprecipations were performed after transfection of Dicer mutants deleted from the N-terminus progressively to encompass the DEAD domain (ΔDEAD), the helicase domain (ΔHelicase), the Domain of Unknown Function 283 (ΔDUF), and the PAZ domain (ΔPAZ) as schematically illustrated in panel B. Cell lysates (lanes 7 to 12) and immunoprecipitations using anti-flag beads were characterized by immunoblotting using anti-flag (upper panel), anti-Tat (middle panel) or anti-Ago2 (bottom panel). **B) **Schematic illustration of the Dicer mutants and summary of the co-immunoprecipitation between Dicer and Tat and Dicer and TRBP.

We next characterized the region in Dicer needed for Tat interaction. Co-immunoprecipation assays were performed using flag-tagged Dicer mutants deleted progressively from the N-terminus to encompass the DEAD domain (ΔDEAD), the Helicase domain (ΔHelicase), the Domain of Unknown Function 283 (ΔDUF), and the PAZ domain (ΔPAZ) (Figure 2A, B) [23]. Each of the mutants expressed well after co-transfection with Tat into 293T cells, and all were immunoprecipitated equivalently using anti-flag beads (IP: anti-flag; lanes 1–6, top panel, Figure 2A). By contrast, when co-immunoprecipitation of Tat was assessed, only wt Dicer and ΔDEAD Dicer mutant (IB: anti-Tat; Figure 2A, middle panel; lanes 2 and 3), but not ΔHelicase, ΔDUF nor ΔPAZ mutants (Figure 2A, lanes 4, 5, 6), associated with Tat. These results suggest that removal of Dicer's helicase domain abolished its ability to co-immunoprecipitate Tat.

We performed two controls for the above experiment. First, we checked that Tat was equally expressed in the lysates of all the transfections (Figure 2A, lanes 8 – 12). Second, we verified that Dicer co-IP'd Ago2. Dicer- Ago2 interaction is dependent on Dicer's RNase III domain located in its C-terminus [24]; and in our experiments, Ago2 co-immunoprecipitated wt Dicer and all the Dicer RNase III domain-containing mutants (Figure 2A; bottom panel, lanes 1 – 6).

We noted with interest that while the interaction of Tat and Dicer is RNA dependent (Figure 1), the presence of Dicer's C-terminal dsRNA binding domain in the above Dicer mutants was insufficient for Dicer to co-immunoprecipitate Tat. Intriguingly, Dicer's helicase domain was previously found to be required to interact with both TRBP and PACT [23]. One interpretation of the collective results is that rather than a simple protein-RNA-protein bridging interaction, there are additional protein-protein contact points between Tat and the helicase region of Dicer which specifies association inside a cell. That Tat, TRBP and PACT all impinge at Dicer's helicase region raises a possibility that these factors may interfere and compete with each other functionally for limiting contact at this locale. Potential competition between Tat and TRBP or Tat and PACT, two key components of miRNA pathway, remains to be further characterized. While under our current experimental conditions no decrease in TRBP recovery was observed after Tat co-IP with Dicer (Figure 1B), whether more notable competition could be seen upon escalated titration of Tat expression remains to be evaluated.

### Tat's trans-activation domain is dispensable for Dicer-association

**Figure 3:**
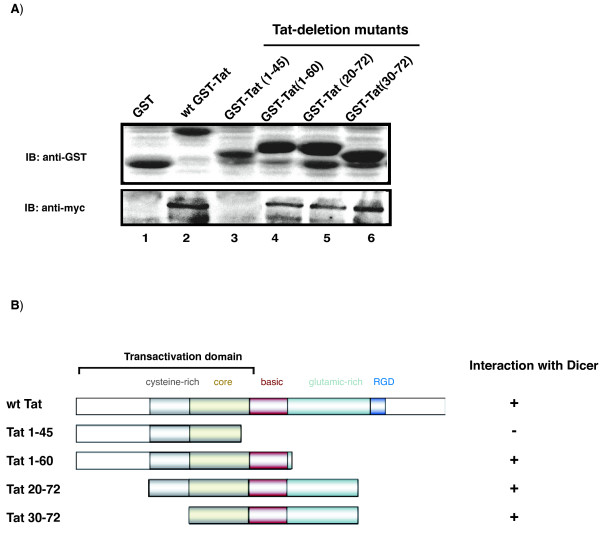
**Tat's transactivation domain Tat (1–45) does not pull-down Dicer from cell lysates**. **A) **Purified GST-Tat and four Tat-deletion mutants, described in B, were used for GST pull down assays of cell lysates from myc-Dicer transfected 293T cells. GST, GST-Tat and GST-Tat mutants were first verified by immunoblotting using anti-GST. The pulled-down of Dicer was analyzed by immunoblotting using anti-myc antibody. **B) **Schematic illustration of Tat mutants and summary of the pull-down results.

We next characterized the region in Tat required to association with Dicer. We performed GST-pull down assays since we had access to a large number of GST-Tat deletion mutants and because our immunoprecipitation of Tat proteins was uneven with differently deleted Tat mutants. Using GST-Tat mutants that included Tat's transactivation domain (Tat 1–45), or Tat's basic region (Tat 1–60), or GST-Tat mutants that were deleted in their transactivation domain but retained their middle regions (Tat 20–72, Tat 30–72; Figure 3B), we assessed pull-down of Dicer using purified GST-Tat and four GST-Tat-deletion mutants. Control GST did not capture Dicer, while GST-Tat (Figure 3A, lane 1), GST-Tat 1–60, GST-Tat 20–72 and GST-Tat 30–72 did pull down Dicer (Figure 3A, lanes 4–6). Interestingly, GST-Tat 1–45 did not pull-down Dicer. These results agree with previous findings that the trans-activation domain of Tat does not account for physical and function interplay with Dicer [25].

Here we have characterized some of the requirements for Tat-Dicer physical association. We found that Tat-Dicer interaction requires RNA, although simple protein-protein bridging by RNA does not seem to be a sufficient explanation. Dicer-Tat interaction also requires Dicer's helicase domain and a portion of Tat's 30–72 amino acids. Whether the latter requirements imply direct protein-protein contact remains to be established.

## List of abbreviations

Ago-2 argonaute 2

miRNA microRNA

RNAi RNA interference

siRNA short interfering RNA

TRBP TAR RNA binding protein

## Competing interests

The author(s) declare that they have no competing interests.

## Authors' contributions

YB carried out the experiments. YB and KTJ conceived of the study and wrote the manuscript.
